# Periorbital Rejuvenation: Artificial Intelligence Highlights the Importance of Brow Lift

**DOI:** 10.1093/asjof/ojae007.011

**Published:** 2024-04-12

**Authors:** Laura Roider, Peter Firouzbakht, Caroline Kreh, Charles Nathan, Christian Prada, Herluf Lund, Deniz Sarhaddi, Kevin Chen

**Affiliations:** Saint Louis University School of Medicine, St. Louis, MO; Saint Louis University School of Medicine, St. Louis, MO; Saint Louis University School of Medicine, St. Louis, MO; St. Louis Cosmetic Surgery, Chesterfield, MO; St. Louis Cosmetic Surgery, Chesterfield, MO; St. Louis Cosmetic Surgery, Chesterfield, MO; St. Louis Cosmetic Surgery, Chesterfield, MO; Saint Louis University School of Medicine, St. Louis, MO

## Abstract

**Goals/Purpose:**

Well described facial changes occur over time, which collectively result in an increased perceived age of an individual. These changes affect everything including the bony framework, facial fat pads, soft tissue, skin quality, and facial muscles. Aesthetic surgery provides an opportunity to reverse the appearance of facial aging.

With the recent increase in mask utilization following the COVID-19 pandemic, an increased focus on the upper face and the impact of age-related changes in the periorbital region has been observed. Blepharoplasty and brow lifts aim to restore a youthful, rested, and attractive appearance by addressing the periorbital region. Blepharoplasty is a commonly performed procedure and has consistently ranked the third most common aesthetic procedure performed by plastic surgeons since 2018. However, following the pandemic we saw a 28% increase in blepharoplasties performed, now totaling 1.4 million in 2022. Over the same time period, the number of brow lifts performed has increased by 60%, now accounting for 352,324 surgeries performed in 2022. Despite this increase, recent surveys demonstrate that nearly 30% of plastic surgeons feel that neuromodulators have completely replaced operative brow lifting procedures. Additionally, a recently published study demonstrated that 62% of patients presenting for blepharoplasty have unfavorable preoperative brow aesthetics. However, only 36% of patients underwent brow lifts.Those with preoperative unfavorable brows who underwent a brow lift procedure were found to have significantly higher postoperative aesthetic scores than those who did not.

Despite the increase in popularity of periorbital procedures, the literature continues to lack objective evaluation and outcome measurements related to perceived facial aging and the impact of individual procedures. The goal of this study is to quantify the impact of different periorbital rejuvenation surgeries on perceived age through the utilization of artificial intelligence (AI) in the form of convolutional neural network algorithms.

**Methods/Technique:**

A retrospective review of patients who underwent periorbital rejuvenation surgery (upper blepharoplasty, lower blepharoplasty, and brow lift) at a single cosmetic practice between 2018-2023 was performed. Charts were reviewed for demographic information, periocular history, prior surgeries, surgical technique utilized, and complications/revisions. Exclusion criteria included: simultaneous procedures, incomplete patient records, poor quality or missing photographs, periocular medical conditions with cosmetic impact, and facial aesthetic procedures performed in the postoperative period.

Pre and post operative frontal photographs of each patient were analyzed using four facial analysis AI platforms (Face++, Betaface, Facelytics, and Everypixel). Each platform generated an age estimate. A collective pre-op and post-op age estimation was then calculated for each patient by averaging the 4 platforms responses. To evaluate accuracy of the AI age estimation, a percent error was calculated by dividing the difference between the true age and estimated preoperative age by the true preoperative age. To account for natural aging between surgery and post-op photographs due to follow-up time, a corrective difference was calculated and subtracted from the mean postoperative estimated age. Statistical analysis included student’s t-test for univariate analysis and linear regression for multivariate analysis. All statistical analyses were performed on Stata version 14.0 (Stata Corporation, College Station, Texas, USA).

**Results/Complications:**

A total of 153 patients were included for analysis, of which 120 underwent upper blepharoplasty, 66 underwent lower blepharoplasty, and 35 underwent a brow lift procedure. The study population consisted of 130 females and 23 males, 95% were Caucasian, and the average preoperative age was 58.1 (± 9.2 years). Post-operative photographs were taken at 3.8 months (± 5.4 months).

Across all AI platforms, the mean age estimation percent error was 10%, with the tendency for AI to underestimate compared to true age. Univariate analysis revealed an overall age reduction following periorbital rejuvenation surgery of 1.03 years (p<0.001), with a standard error of 0.257. When controlling for all other procedures via linear regression, patients who underwent brow lifts demonstrated a statistically significant age reduction, with the mean AI perceived age reduction of 1.72 years (p=0.007). No significant age reduction was identified for patients undergoing lower blepharoplasty (regardless of technique), ptosis repair, or fat pad resection.

**Conclusion:**

The results of this study highlight the rejuvenation power of a brow lift, with an average age reduction of nearly 2 years. In our patient population, brow lifting procedures conveyed the most age reduction of periorbital surgery. Recently published literature indicates that brow lifts are under performed and can serve as a potential avenue for further facial rejuvenation. Therefore, when planning periorbital rejuvenation, a thorough preoperative evaluation of brow position and shape should be performed and additional consideration should be given to operative brow lifting procedures.

